# Does provider-initiated HIV testing and counselling lead to higher HIV testing rate and HIV case finding in Rwandan clinics?

**DOI:** 10.1186/s12879-016-1355-z

**Published:** 2016-01-25

**Authors:** Felix R. Kayigamba, Daniëla Van Santen, Mirjam I. Bakker, Judith Lammers, Veronicah Mugisha, Emmanuel Bagiruwigize, Ludwig De Naeyer, Anita Asiimwe, Maarten F. Schim Van Der Loeff

**Affiliations:** 1 INTERACT, Kigali, Rwanda; 2 KIT Biomedical Research, Royal Tropical Institute (KIT), Amsterdam, The Netherlands; 3 Academic Medical Center (AMC), Amsterdam, The Netherlands; 4 ICAP, Mailman School of Public Health, Columbia University, Kigali, Rwanda; 5 Ruhengeri hospital, Ministry of Health, Kigali, Rwanda; 6 University of Rwanda, Kigali, Rwanda; 7 Amsterdam Institute of Global Health and Development (AIGHD), Academic Medical Center (AMC), Amsterdam, The Netherlands; 8 Center for Infection and Immunity Amsterdam (CINIMA), AMC, Amsterdam, The Netherlands; 9 Public Health Service of Amsterdam (GGD), Amsterdam, The Netherlands

**Keywords:** HIV testing rate, HIV case finding, PITC, Rwanda

## Abstract

**Background:**

Provider-initiated HIV testing and counselling (PITC) is promoted as a means to increase HIV case finding. We assessed the effectiveness of PITC to increase HIV testing rate and HIV case finding among outpatients in Rwandan health facilities (HF).

**Methods:**

PITC was introduced in six HFs in 2009-2010. HIV testing rate and case finding were compared between phase 1 (pre-PITC) and phase 3 (PITC period) for outpatient-department (OPD) attendees only, and for OPD and voluntary counseling & testing (VCT) departments combined.

**Results:**

Out of 26,367 adult OPD attendees in phase 1, 4.7 % were tested and out of 29,864 attendees in phase 3, 17.0 % were tested (*p* < 0.001). The proportion of HIV cases diagnosed was 0.25 % (67/26,367) in phase 1 and 0.46 % (136/29864) in phase 3 (*p* < 0.001). In multivariable analysis, both testing rate and case finding were significantly higher in phase 3 for OPD attendees. In phase 1 most of the HIV testing was done in VCT departments rather than at the OPD (78.6 % vs 21.4 % respectively); in phase 3 this was reversed (40.0 % vs 60.0 %; *p* < 0.001). In a combined analysis of VCT and OPD attendees, testing rate increased from 18.7 % in phase 1 to 25.4 % in phase 3, but case finding did not increase. In multivariable analysis, testing rate was significantly higher in phase 3 (OR 1.67; 95 % CI 1.60-1.73), but case finding remained stable (OR 1.09; 95 % CI 0.93-1.27).

**Conclusion:**

PITC led to a shift of HIV testing from VCT department to the OPD, a higher testing rate, but no additional HIV case finding.

**Electronic supplementary material:**

The online version of this article (doi:10.1186/s12879-016-1355-z) contains supplementary material, which is available to authorized users.

## Background

Sub-Saharan Africa has the greatest burden of HIV with 25 million people living with HIV by the end of 2012 [1]. Knowledge of HIV status is imperative for prevention and timely start of HIV care [2–4]. About 30 % of people in sub-Saharan countries have never been tested for HIV [1]. To achieve universal HIV testing, the World Health Organization (WHO) recommends provider initiated testing and counselling (PITC) to facilitate timely diagnosis and access to HIV related services [2]. According to this policy all patients presenting at health facilities (HFs) in generalized HIV epidemics, regardless of signs or symptoms, should be offered an HIV test on an opt-out basis, making it a standard component of medical care [2].

PITC has mainly been implemented in antenatal (ANC) and tuberculosis (TB) clinics in sub-Saharan countries, with high overall testing rate levels [5–12]. Studies from several countries (Kenya, Ethiopia, Uganda) have reported high levels of acceptability of PITC, increased HIV testing rate, and linkage to care after HIV diagnosis in outpatient departments (OPDs) [13–16]. Rwanda is a country with a generalised epidemic [1], but the adult HIV prevalence (3 %) [17] is low compared to that of most sub-Saharan African countries. Rwanda has a dense network of health facilities that offer HIV testing and antiretroviral treatment, and the coverage of cART (80 %) is higher than in most countries in the region [18].

The Rwanda Ministry of Health (MOH) adopted PITC as a policy to increase the opportunity for HIV testing and ensure timely HIV diagnosis among HF attendees. We implemented PITC in 2009/2010 and assessed whether PITC was an effective strategy to increase HIV testing rate and HIV case finding in outpatient departments of six Rwandan HFs. In order to examine whether PITC led to a shift from testing at voluntary counseling and testing (VCT) clinics to testing at OPDs, we also collected data on testing at VCT departments.

## Methods

### Setting

Four HFs in Musanze district (North-West Rwanda) and four HFs in Gasabo district (Central Rwanda; area of the capital) were purposefully selected for this study, ensuring inclusion of urban and rural HFs with sufficient numbers of attendees. All included HFs had a complete range of HIV testing, care and treatment services.

### Study design

The study consisted of three phases: in phase 1 (routine care period; March-May 2009) PITC was not operational; in phase 2 (preparation phase; June-November 2009) PITC was introduced; and in phase 3 (PITC intervention period; December 2009-February 2010) PITC was operational. PITC was introduced in six HFs while two served as controls.

### Intervention

Biomedical materials for PITC were delivered to six HFs. Health care workers (HCWs) were trained to offer PITC, administer HIV testing, and use registers adapted for the study to record testing. In TB and ANC departments PITC was already commonly practiced prior to the start of the study, following national policy. With the exception of Ruhengeri hospital, VCT departments were operational in the other HFs, where attendees could go and be tested on their own accord, or where they could be referred to by health staff from other departments. During phase 3, PITC was newly operational in the OPD. HCWs informed clinic attendees that it was MOH-recommended policy to offer HIV testing to all clients [19]. PITC was offered in three ways. Option 1 involved a rapid test by the HCW using a finger-prick blood sample in the consultation department. Option 2 involved a test on a venous blood sample drawn by the HCW and sent to the laboratory for rapid testing. Option 3 involved the HCW offering the test, and upon consent sending the attendee to the laboratory for a venous blood draw and rapid testing. In all options, HCWs provided post-test counseling. Each option was implemented at two intervention sites (Table 1).

**Table 1:** Characteristics of health facilities included in the PITC study, Rwanda 2009-10

Study site	Province	Level of urbanization	Type	Total attendees^a^	Intervention^b^	Remarks
Rwaza	Northern	Rural	Health center	7,147	Option 1	HF staff started some form of PITC already prior to phase 1
Kinyinya	Kigali City	Urban	Health center	7,964	Option 1	
Ruhengeri	Northern	Semi-urban	Hospital	9,191	Option 2	No VCT department at this HF; clients were referred to Muhoza
Muhoza	Northern	Semi-urban	Health center	26,125	Option 2	HF located next to Ruhengeri hospital (500 m).
Kibagabaga	Kigali City	Urban	Hospital	6,771	Option 3	This hospital was affected by managerial changes during phase 3
Kimironko	Kigali City	Urban	Health center	13,135	Option 3	
Gasiza (c)	Northern	Rural	Health center	7,535	Control	
Kabuye (c)	Kigali City	Semi-urban	Health center	6,980	Control	Staff initiated some form of PITC during phase 3 on their own initiative.

^a^Total attendees: people that sought health services in all departments (Antenatal Care, Family Planning, Out-patients department, Tuberculosis department, Voluntary counseling and testing) at the study sites during the study periods, March-May 2009 and December 2009-February 2010

^b^Option 1 involved a rapid test by the health care worker (HCW) using a finger-prick blood sample in the consultation department. Option 2 involved a test on a venous blood sample drawn by the HCW and sent to the laboratory for rapid testing. Option 3 involved the HCW offering the test, and upon consent sending the attendee to the laboratory for a venous blood draw and rapid testing. In all options, HCWs provided post-test counseling

Abbreviations: *HF* Health Facility, *PITC* Provider Initiated testing and Counseling, *VCT* Voluntary Counseling and testing

### HIV testing

Both in the routine care and the intervention period, a serial rapid test algorithm was used to test for HIV. The first test used in the rapid test algorithm was Determine HIV-1/2/O (Abbott Laboratories, Abbott Park, Illinois, USA). If the Determine was negative, no further testing was done and the patient was diagnosed as HIV negative. If positive, the UniGold (Trinity Biotech, Bray, Ireland) test was done. In case Determine and UniGold had discrepant results, a third test, the Capillus HIV-1/HIV-2 (Cambridge Biotech Corp. Worcester, MA, USA), was done. Capillus acted as a tiebreaker in order to reach a final HIV result.

### Data abstraction

Trained field workers abstracted data from registers in the laboratory, OPD and VCT using structured forms in phase 1 and phase 3. To enable linkage between registers in different departments in phase 1, some adaptations were made to the registers. During phase 2 registers from the OPD were adapted to capture additional data on PITC implementation. The variables introduced to the existing registers were: HIV test offer, test acceptance, reason for refusal, test result received, history of fever and number of HIV tests done in the preceding two years. To facilitate linkage between registers from different departments, patient reference numbers on stickers were inserted in the registers starting from the initial point of entry into the clinic. The following data were abstracted: age, sex, study site, tested, and test result. Data on test offer, test acceptance, reasons for refusal, receipt of result, history of fever and number of previous HIV tests in preceding 2 years were abstracted only during phase 3 in the OPD.

### Outcome measures

Our study had two primary outcomes: HIV testing rate and HIV case finding. HIV testing rate was defined as the proportion of individuals tested for HIV out of the total number of attendees of a department. HIV case finding was defined as the proportion of individuals that were diagnosed with HIV, out of the total number of attendees of a department. Secondary outcome measures were test offer, test acceptance, test result received and reasons for refusing an HIV test during the intervention phase. A “test offer” consisted of explicitly stating to a patient that an HIV test would be done unless he/she declined. “Test acceptance” indicates that the patient consented to be tested for HIV after having been offered a test. “Test result received” means the patient obtained the result of the HIV test after being tested. If clinic attendees declined to be tested for HIV, they were asked for the reason to decline (“reasons for refusal”).

### Data management and analysis

Our analyses were limited to attendees aged 15 years or above. The characteristics of the HF attendees were described using percentages, medians and interquartile ranges [IQR]. Multivariable logistic regression models were used to establish whether PITC (i.e. phase 3) was an independent determinant for the two main outcomes, HIV testing rate and HIV case finding, when adjusted for age and sex of attendees, and study site. Robust standard errors were used to take account of clustering effect of site. P values of <0.05 were considered statistically significant. For all analyses Stata version 11.2 was used (Stata Corp; College Station, TX, USA).

### Ethics

The Rwanda National Ethics Committee and the research committee of the Academic Medical Center, Amsterdam, provided approval for this study. All patients were aware that as part of clinical care their demographic and clinical data were registered in paper-based clinic registers. As only such routinely collected data were abstracted from clinic registers, and no names were entered into the electronic study database, the ethics committees did not require that written informed consent was sought from patients.

## Results

### Characteristics of OPD attendees

During phase 1 of the study (March-May 2009), a total of 31,204 adult attendees were registered at the OPDs of the eight HFs included in this study. The majority (64 %) were women, and the median age was 28 years (IQR 22–41). The number of attendees ranged from 1,878 in Kabuye to 10,745 in Muhoza (Additional file 1: Table S1). At the same HFs, 34,512 adult attendees were registered during phase 3 (December 2009-February 2010). The median age of OPD attendees in phase 3 was also 28 years (IQR 22–40) and a similar proportion was female (61 %). Again Kabuye registered the smallest number of attendees (2,137) and Muhoza the largest number (8,147) (Additional file 1: Table S1).

### HIV testing rate at OPD

**Fig. 1:**
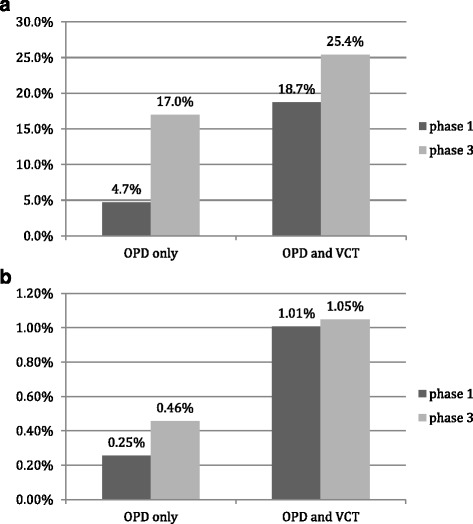
**a**. HIV testing rate at OPD. **b**. HIV case finding at OPD

Out of 26,367 attendees in phase 1 at the six intervention sites, 4.7 % (1,234) were tested for HIV. During phase 3, out of 29,864 attendees 17.0 % (5,065) tested for HIV (Table 2, Fig. 1a). The difference in testing rate between the phases was 12.3 % (*p* < 0.001). Important differences were observed between the sites: in Muhoza and Kimironko testing rate increased with 20.2 % and 15.9 % respectively during phase 3, but in Kibagabaga the testing rate decreased with 7.8 %. Rwaza had the highest testing rate in both phase 1 and phase 3. Here, clinic staff had introduced a form of PITC on their own initiative prior to phase 1.

**Table 2:** HIV testing rate and HIV case finding during phase 1 and phase 3 by study site in outpatient departments in eight health facilities, PITC study, Rwanda 2009-2010

	Phase 1						Phase 3						Differences between phases 3 and 1	
Study site	N	HIV testing rate n	HIV testing rate %	HIV+ n	HIV+ % (of all tested)	HIV+ % (of all eligible)	N	HIV testing rate n	HIV testing rate %	HIV+ n	HIV+ % (of all tested)	HIV+ % (of all eligible)	Difference in testing rate	Difference in HIV case finding
	A	B	C = B/A	D	E = D/B	F = D/A	G	H	I = H/G	J	K = J/H	L = J/G	M = I - C	N = L - F
Rwaza	2,270	615	27.1 %	10	1.6 %	0.44 %	3,654	1,255	34.3 %	3	0.24 %	0.08 %	7.3 %	-0.36 %
Kinyinya	2,584	34	1.3 %	6	17.6 %	0.23 %	3,488	311	8.9 %	19	6.1 %	0.54 %	7.6 %	0.31 %
Ruhengeri	3,829	195	5.1 %	8	4.1 %	0.21 %	5,259	612	11.6 %	11	1.8 %	0.21 %	6.5 %	0.00 %
Muhoza	10,745	50	0.5 %	6	12.0 %	0.06 %	8,147	1,682	20.6 %	47	2.8 %	0.58 %	20.2 %	0.52 %
Kibagabaga	2,131	313	14.7 %	30	9.6 %	1.41 %	3,465	240	6.9 %	11	4.6 %	0.32 %	-7.8 %	-1.09 %
Kimironko	4,808	27	0.6 %	7	25.9 %	0.15 %	5,851	965	16.5 %	45	4.7 %	0.77 %	15.9 %	0.62 %
*Total*	26,367	1,234	4.7 %	67	5.4 %	0.25 %	29,864	5,065	17.0 %	136	2.7 %	0.46 %	12.3 %	0.21 %
Gasiza (c)	2,959	41	1.4 %	4	9.8 %	0.14 %	2,511	22	0.9 %	4	18.2 %	0.16 %	-0.5 %	0.02 %
Kabuye (c)	1,878	201	10.7 %	5	2.5 %	0.27 %	2,137	605	28.3 %	13	2.1 %	0.61 %	17.6 %	0.34 %
*Total*	4,837	242	5.0 %	9	3.7 %	0.19 %	4,648	627	13.5 %	17	2.7 %	0.37 %	8.5 %	0.18 %

HIV + = HIV positive individuals. (c) Control site

In the two control sites, testing rate increased from 5.0 % to 13.5 % (*p* < 0.001). One control site (Gasiza) did not experience a change in testing rate, but the other site (Kabuye) experienced an increase in testing rate of 17.6 % (Table 2). In this latter clinic the staff initiated a form of PITC on their own initiative between phase 1 and 3. In multivariable analysis (adjusting for age, sex and site), an HIV test was significantly more likely to be done in phase 3 compared to phase 1 in both the intervention (adjusted Odds Ratio [aOR] 4.57; 95 % Confidence Interval [CI] 4.26–4.90; Table 3) and control sites (aOR 2.76; 95 % CI 2.34–3.24; Additional file 1: Table S2).

**Table 3:** Multivariable logistic regression to determine the association of the PITC intervention with HIV testing rate and HIV case finding in the outpatient department of 6 health facilities (upper panel) and in the outpatient and voluntary counseling and testing departments combined of the same 6 health facilities (lower panel); Rwanda, 2009-2010

	HIV testing rate				HIV case finding			
OPD only	n/N (%)	aOR	(95 % CI)	*P*	n/N (%)	aOR	(95 % CI)	*P*
Study phase								
Phase 1	1,234/26,367 (4.7 %)	1		<0.001	67/26,367 (0.25 %)	1		<0.001
Phase 3	5,065/29,864 (17 %)	4.57	(4.26-4.90)		136/29,864 (0.46 %)	1.97	(1.41-2.76)	
Study site								
Ruhengeri	807/9,088 (8.9 %)	1		<0.001	19/9,088 (0.21 %)	1		<0.001
Muhoza	1,732/18,892 (9.2 %)	1.18	(1.08-1.30)		53/18,892 (0.28 %)	1.87	(1.06-3.31)	
Kibagabaga	553/5,596 (9.9 %)	0.79	(0.69-0.89)		41/5,596 (0.73 %)	3.10	(1.69-5.68)	
Kinyinya	345/6,072 (5.7 %)	0.55	(0.48-0.63)		25/6,072 (0.41 %)	2.32	(1.24-4.35)	
Kimironko	992/10,659 (9.3 %)	0.97	(0.88-1.08)		52/10,659 (0.49 %)	3.13	(1.78-5.51)	
Rwaza	1,870/5,924 (31.6 %)	4.65	(4.21-5.13)		13/5,924 (0.22 %)	1.19	(0.57-2.49)	
Sex								
Females	3,685/34,610 (10.7 %)	1		<0.001	111/34,610 (0.32 %)	1		0.078
Males	2,597/21,035 (12.4 %)	1.19	(1.13-1.26)		92/21,035 (0.44 %)	1.30	(0.98-1.73)	
Age group (years)								
15 - 24	2,275/19,643 (11.6 %)	1		<0.001	35/19,643 (0.18 %)	1		<0.001
25 - 34	1,732/16,687 (10.4 %)	0.93	(0.87-1.00)		65/16,687 (0.39 %)	2.09	(1.38-3.16)	
35 - 44	863/7,648 (11.3 %)	0.92	(0.84-1.01)		48/7,648 (0.63 %)	3.73	(2.41-5.78)	
≥45	1,270/11,470 (11.1 %)	0.77	(0.71-0.83)		42/11,470 (0.37 %)	2.38	(1.52-3.71)	
OPD & VCT combined	n/N (%)	aOR	(95 % CI)	*P*	n/N (%)	aOR	(95 % CI)	*P*
Study phase								
Phase 1	5,779/30,914 (18.7 %)	1		<0.001	311/30,914 (1.01 %)	1		<0.001
Phase 3	8,442/33,242 (25.4 %)	1.67	1.60-1.73		348/33,242 (1.05 %)	1.09	0.93-1.28	
Study site								
Ruhengeri	807/9,088 (8.9 %)	1		<0.001	19/9,088 (0.21 %)	1		<0.001
Muhoza	6,673/23,833 (28.0 %)	3.95	3.65-4.28		270/23,833 (1.13 %)	6.26	3.78-10.38	
Kibagabaga	959/6,004 (16.0 %)	1.49	1.34-1.66		104/6,004 (1.73 %)	8.27	4.87-14.06	
Kinyinya	1,223/6,950 (17.6 %)	1.91	1.73-2.10		94/6,950 (1.35 %)	7.22	4.25-12.28	
Kimironko	1,763/11,431 (15.4 %)	1.58	1.44-1.73		153/11,431 (1.34 %)	7.39	4.41-12.38	
Rwaza	2,796/6,850 (40.8 %)	7.04	6.43-7.70		19/6,850 (0.28 %)	1.57	0.81-3.06	
Sex								0.417
Females	8,242/39,170 (21.0 %)	1		<0.001	413/39,170 (1.05 %)	1		
Males	5,959/24,397 (24.4 %)	1.34	1.29-1.39		246/24,397 (1.01 %)	0.93	0.80-1.10	
Age group (years)								
15 - 24	5,498/22,867 (24.0 %)	1		<0.001	157/22,867 (0.69 %)	1		<0.001
25 - 34	4,728/19,685 (24.0 %)	1.11	1.06-1.16		270/19,685 (1.37 %)	2.02	1.65-2.46	
35 - 44	1,887/8,672 (21.8 %)	0.91	0.85-0.96		128/8,672 (1.48 %)	2.42	1.91-3.06	
≥45	1,933/12,133 (15.9 %)	0.54	0.51-0.58		90/12,133 (0.74 %)	1.34	1.03-1.73	

Abbreviations: *aOR* adjusted Odds ratio, *CI* confidence interval, *OPD* outpatient department, *VCT* voluntary counselling and testing

Upper panel, OPD only data; information on age was missing from 783 attendees and information on sex from 586 attendees

Lower panel, OPD & VCT data combined: information on age was missing from 799 attendees and information on sex from 589 attendees

Adjusted odds ratios are adjusted for the other variables in the Table

During phase 3, out of 29,863 eligible OPD attendees in the intervention sites, 92.9 % (27, 753) were offered an HIV test, 19.6 % of those accepted; of those who accepted 93.3 % were tested, of whom 92.2 % received results (Additional file 1: Figure S1). Among the 27,753 attendees in phase 3 who were offered an HIV test and who declined, a reason for refusal was noted for 5,876 (21.2 %). The most common reasons reported for refusing an HIV test were previous knowledge of HIV status (33.4 %; 1,963/5,876) and lack of interest (25.3 %; 1,486/5,876) (Additional file 1: Figure S2).

### HIV case finding at OPD

The proportion of HIV positive attendees identified in phase 1 at the intervention sites was 0.25 % (67/26,367) while it was 0.46 % (136/29,864) during phase 3, an absolute increase of 0.21 % (*p* < 0.001; Table 2, Fig. 1b). Important differences were seen between sites. Rwaza and Kibagabaga experienced a decrease in HIV case finding. Ruhengeri did not show a change in case finding and Muhoza and Kimironko showed an increase of 0.52 % and 0.62 % respectively. In multivariable analysis (adjusting for age, sex and site), HIV case finding in the intervention sites was significantly higher in phase 3 (aOR 1.97; 95 % CI 1.41–2.76) compared to phase 1 (Table 3).

In the control sites the percentage of HIV positive attendees increased by 0.18 % (from 0.19 % to 0.37 %; *p* = 0.094). There was no change in Gasiza and an increase of 0.34 % in Kabuye. The aOR for the control sites in phase 3 was 1.85 (95 % CI 0.83–4.12) (Additional file 1: Table S2) compared to phase 1.

### Characteristics of OPD and VCT attendees

In a secondary analysis we examined HIV testing rate and case finding in the combined attendee populations of OPD and VCT departments. The VCT departments of the eight health facilities registered 6,541 new clients in phase 1 and 5,129 in phase 3. During phase 1, a total of 37,747 adult attendees were registered at the OPD and VCT departments of the eight HFs (Additional file 1: Table S3). The majority (63 %) were women, and the median age was 28 years (interquartile range [IQR] 22–40). The number of attendees ranged from 2,381 in Kibagabaga to 13,614 in Muhoza. At the same HFs, 39,642 adult attendees were registered during phase 3. The median age of attendees in phase 3 was also 28 years (IQR 22–39) and a similar proportion was female (60 %). Gasiza registered the smallest number of attendees (3,154) and Muhoza the largest number (10,219) (Additional file 1: Table S3).

### HIV testing rate at OPD and VCT combined

Out of 30,914 attendees at OPDs and VCT departments in phase 1 at the six intervention sites, 18.7 % (5,779) were tested for HIV; during phase 3, out of 33,242 attendees, 25.4 % (8,442) were tested for HIV (*p* < 0.001, Table 4, Fig. 1a). In phase 1, 78.6 % of tested attendees (4,545/5779) were tested at the VCT; in phase 3 this had declined to 40.0 % (3,377/8,442; *p* < 0.001). So there was both a relative and an absolute decline in the number of attendees tested at VCT departments.

**Table 4:** HIV testing rate and HIV case finding during phase 1 and 3 by study site in outpatient and voluntary counseling and testing departments in eight health facilities, PITC study, Rwanda 2009-2010

	Phase 1						Phase 3						Differences between phases 3 and 1	
Study site	N	HIV testing rate n	HIV testing rate %	HIV + n	HIV+ % (% of testing rate)	HIV+ % (% of eligible)	N	HIV testing rate n	HIV testing rate %	HIV+ n	HIV+ % (% of testing rate)	HIV+ % (% of eligible)	Difference in testing rate	Difference in HIV case finding
	A	B	C = B/A	D	E = D/B	F = D/A	G	H	I = H/G	J	K = J/H	L = J/G	M = I - C	N = L - F
Rwaza	2,863	1208	42.2 %	14	1.2 %	0.49 %	3,987	1,588	39.8 %	5	0.31 %	0.13 %	-2.4 %	-0.36 %
Kinyinya	3,072	522	17.0 %	30	5.7 %	0.98 %	3,878	701	18.1 %	64	9.1 %	1.65 %	1.1 %	0.67 %
Ruhengeri	3,829	195	5.1 %	8	4.1 %	0.21 %	5,259	612	11.6 %	11	1.8 %	0.21 %	6.5 %	0.00 %
Muhoza	13,614	2919	21.4 %	128	4.4 %	0.94 %	10,219	3,754	36.7 %	142	3.8 %	1.39 %	15.3 %	0.45 %
Kibagabaga	2,381	561	23.6 %	72	12.8 %	3.02 %	3,623	398	11.0 %	32	8.0 %	0.88 %	-12.6 %	-2.14 %
Kimironko	5,155	374	7.3 %	59	15.8 %	1.14 %	6,276	1389	22.1 %	94	6.8 %	1.50 %	14.88 %	0.35 %
Total	30,914	5,779	18.7 %	311	5.4 %	1.01 %	33,242	8,442	25.4 %	348	4.1 %	1.05 %	6.70 %	0.04 %
Gasiza (c)	3,602	684	19.0 %	16	2.3 %	0.44 %	3,154	665	21.1 %	5	0.8 %	0.16 %	2.09 %	-0.29 %
Kabuye (c)	3,231	1554	48.1 %	35	2.3 %	1.08 %	3,246	1714	52.8 %	60	3.5 %	1.85 %	4.71 %	0.77 %
Total	6,833	2,238	32.8 %	51	2.3 %	0.75 %	6,400	2,379	37.2 %	65	2.7 %	1.02 %	4.42 %	0.27 %

Abbreviations: HIV+: HIV positive individuals, *c* control

In multivariable analysis (adjusting for age, sex and site), the testing rate was significantly increased in phase 3 in both the intervention (aOR 1.67; 95 % CI 1.60–1.73; Table 3) and control sites (aOR 1.14; 95 % CI 1.06–1.24; Additional file 1: Table S4), compared to the rate in phase 1.

### HIV case finding at OPD and VCT combined

The proportion of HIV positive attendees identified in phase 1 at intervention sites was 1.01 % (311/30,914) while it was 1.05 % (348/33,242) during phase 3, an increase of 0.04 % (*p* = 0.608; Table 4, Fig. 1b). In multivariable analysis (adjusting for age, sex and study site), case finding was not significantly increased in phase 3 in intervention sites (aOR 1.09; 95 % CI 0.93–1.28) compared to phase 1 (Table 3).

In the control sites the percentage of HIV positive attendees increased by 0.27 % (from 0.75 % to 1.02 %; *p* = 0.094). The aOR for HIV case finding at the control sites in phase 3 was 1.29 (95 % CI 0.89–1.86) compared to phase 1 (Additional file 1: Table S4).

## Discussion

### Main findings

PITC was associated with a higher HIV testing rate among attendees of OPDs of six Rwandan HFs, but the increase in HIV case finding was limited. The increase in HIV testing at the OPD was accompanied by a decrease in HIV testing at the VCT departments. In a combined analysis of attendees of OPD and VCT departments, PITC was associated with an increase in HIV testing rate but not with additional HIV case finding. The findings from our study may be applicable to other settings with a similar HIV epidemiologic profile, such as a relatively low prevalence and high cART coverage, and thus may inform decision-making in Rwanda and elswhere.

### HIV testing rate

The implementation of PITC in the OPD led to a 4.5 fold increase in testing rate during the intervention phase compared to routine care period when studying the OPD only. A combined analysis of the OPD and VCT showed a 1.7 fold increase in testing rate. Our data indicate a shift of testing from VCT to OPD after the introduction of PITC: both the absolute number of HIV tests done at the VCT declined, and the proportion of HIV tests done at the VCT (from 79 % to 40 %). This may indicate that patients who were tested in phase 3 at OPD clinics might have been tested in the VCT department in the absence of PITC. Alternatively, perhaps patients preferred to be tested in the OPD to avoid stigma associated with VCT departments [20]. A study from Zambia found that introducing PITC in nine OPDs directly increased HIV testing rates compared with the number tested under VCT in the same month; so PITC provided an additional testing route rather than replacing VCT [15]. A study from Botswana reported an increase of HIV testing rate following the nationwide introduction of PITC [21]. A study from Gauteng Province, South Africa reported a 2.9 fold increase in HIV testing rate under PITC compared to VCT referral model [16]. Another study from Durban, South Africa, compared a period of standard care to a subsequent period when all patients registered at the OPD were given an educational intervention and offered a rapid HIV test as a routine; both HIV testing rate and HIV case finding increased strongly (5-fold higher weekly HIV case finding) [22]. This report did not mention the impact of the intervention on the total number of VCT clients/tests, and whether a similar shifting phenomenon occurred as in our setting.

Attendees who declined testing during the intervention phase were asked by health care workers for the reasons for refusing an HIV test. In line with Cunningham et al. previous knowledge of HIV status and perceived lack of interest were the most common reasons for refusing an HIV test [23]. Previous knowledge as one of the common reasons for declining may imply that relatively many people were aware of their HIV status. This finding must be seen in the context of results from a recent global report on sub-Saharan African countries, including Rwanda, which indicated that in Rwanda an estimated 39 % of people were tested within the last year and thus are likely to know their HIV status [1]. The Rwandan demographic and health survey reported similar findings (39 % of women and 38 % of men received results from an HIV test taken during the 12 months prior to the survey) [17]. A South African study reported that 31 % declined testing because they were uncomfortable or afraid of an HIV test and 19 % reported not feeling the need to be tested [16]. Although OPD attendees did not incur costs to get tested in our study, a Ugandan study reported that the cost of testing and lack of perceived risk were the reasons attributed to prior lack of testing [24]. Strategies such as intensifying health education for patients that aim to remove misconceptions about HIV are needed [25]. The importance of retesting despite previous negative tests should be emphasized during post-test counseling. Another strategy may be to establish a stigma-free and enabling environment at health facilities by re-arranging the patient flow and fully integrating HIV testing into other services, instead of stand-alone HIV clinics or laboratories.

### HIV case finding

Increasing HIV case finding is critical towards achieving universal access to care and treatment services and PITC could be an effective approach [15]. In our study, the absolute difference in HIV case finding between phase 3 and phase 1 was 0.21 % in the OPD, which represents a limited increase. There were important differences seen between sites; some had a limited increase while others experienced a decrease in case finding. In one health facility (Kibagabaga) this decrease was explained by the decrease of HIV testing rate due to management changes at this site. In another facility (Rwaza) this decrease might be explained by the already high HIV testing rate in phase 1. The analysis of the combined VCT and OPD populations demonstrated that PITC was not significantly associated with increased HIV case finding. In Zambia, the integration of PITC into routine OPD care substantially increased case finding of HIV positive patients [15, 26]. Systematic reviews have demonstrated the importance of PITC in identifying undiagnosed HIV infections [27, 28]. Studies have also reported other benefits of routine HIV testing for identification of previously undiagnosed HIV positive cases, i.e. increased knowledge of HIV status and reduction of risk behavior [14, 22, 29, 30]. So our data are different from findings from other published studies regarding the effect of PITC on HIV case finding. The differences in HIV prevalence and the level of HIV services delivery may explain this discrepancy. Rwanda has a relatively low prevalence [17], a decreasing trend of new infections and a high ART coverage (above 80 %) compared to the countries in which other PITC studies were done [18]. PITC led to an increase in the HIV testing rate, but not to an increase in case finding. This would suggest that in these settings PITC at general OPDs is not contributing to reaching the 90-90-90 goals.

### Limitations

Our study had some limitations. The study was conducted in eight clinics that were purposely chosen to be different in several respects (urban-rural, relatively high vs. relatively low HIV prevalence, small versus very large clinics). Rwanda is a relatively small country with rather similar levels of health services provision across the nation, albeit it with some variation between urban and rural settings. Therefore we believe the results may be applied to national scale. We implemented a before-and-after-intervention study design; this lacks the strengths of a randomized clinical trial and therefore the study results should be interpreted with caution. Although we introduced and operationalized PITC, the implementation was not fully rigorous; health workers reported that they had offered an HIV test to 93 % of the attendees and that 19 % of these accepted the test. The variation between sites in characteristics and result did not allow to draw conclusions regarding the three different testing options. Phase 3, during which PITC was implemented was a different season than phase 1; this may have affected the composition of the group of attendees, and hence testing history and willingness to be tested. However, seasonal effects have a larger impact on the composition of pediatric clinic populations than on that of adult clinic populations. As our study population was limited to those aged 15 years or above, we think seasonal effects have not played a large role in the observed differences.

## Conclusions

PITC led to an increase in HIV testing rate and a limited additional HIV case finding among OPD attendees at intervention sites compared with the routine care period. Previous knowledge of HIV status and perceived lack of interest were the most common reasons to decline testing. Introducing PITC in the OPDs led to a shift of HIV testing from the VCT department to the OPD at intervention sites. HIV testing is important in prevention, and PITC may play a large role here. However, in our setting PITC was not effective in increasing HIV case finding. We recommend further research taking into consideration differences in HIV prevalence, population HIV testing rates, and ART coverage.

## Additional file

Additional file 1: Table S1. Characteristics of 65,716 clinic attendees of outpatient departments in eight health facilities by study phase and site, PITC study, Rwanda 2009–2010. **Table S2.** Multivariable logistic regression to determine the association of study phase with HIV testing rate and HIV case finding in the outpatient department of 2 control health facilities in Rwanda, 2009–2010. **Table S3.** Characteristics of 77,389 clinic attendees of outpatient and voluntary counseling and testing departments in eight health facilities by study phase and site, PITC study, Rwanda 2009–2010. **Table S4.** Multivariable logistic regression to determine the association of study phase with HIV testing rate and HIV case finding in the outpatient and voluntary counseling and testing departments of 2 control health facilities in Rwanda, 2009–2010. **Figure S1.** HIV testing rate and testing cascade in OPD in phase 3 at intervention sites, Rwanda, 2009–10. **Figure S2.** Reasons for refusing an HIV test in phase 3 at intervention sites, Rwanda, 2009–10. (DOC 311 kb)

## Abbreviations

ANC: Antenatal care
aOR: Adjusted Odds Ratio
cART: Combination antiretroviral therapy
CI: Confidence Interval
HCW: Health care worker
HF: Health facility
HIV: Human Immunodeficiency virus
IQR: Interquartile ranges
MOH: Ministry of health
OPD: Outpatient department
PITC: Provider-initiated HIV testing and counselling
TB: Tuberculosis
VCT: Voluntary counselling and testing
WHO: World health organization
