# Supplementary material for: Does provider-initiated HIV testing and counselling lead to higher HIV testing rate and HIV case finding in Rwandan clinics?
Source: BMC Infect Dis. 2016 Jan 25;16:26. doi: 10.1186/s12879-016-1355-z (PMC4727293; doi:10.1186/s12879-016-1355-z)
Supplement: Additional file 1: Table S1. — Characteristics of 65,716 clinic attendees of outpatient departments in eight health facilities by study phase and site, PITC study, Rwanda 2009–2010. Table S2. Multivariable logistic regression to determine the association of study phase with HIV testing rate and HIV case finding in the outpatient department of 2 control health facilities in Rwanda, 2009–2010. Table S3. Characteristics of 77,389 clinic attendees of outpatient and voluntary counseling and testing departments in eight health facilities by study phase and site, PITC study, Rwanda 2009–2010. Table S4. Multivariable logistic regression to determine the association of study phase with HIV testing rate and HIV case finding in the outpatient and voluntary counseling and testing departments of 2 control health facilities in Rwanda, 2009–2010. Figure S1. HIV testing rate and testing cascade in OPD in phase 3 at intervention sites, Rwanda, 2009–10. Figure S2. Reasons for refusing an HIV test in phase 3 at intervention sites, Rwanda, 2009–10. (DOC 311 kb) [file 12879_2016_1355_MOESM1_ESM.doc]

**Supplement to:**

**Does provider-initiated HIV testing and counselling lead to higher HIV** testing rate **and HIV case finding in Rwandan clinics?**

**Authors:**

Felix R Kayigamba^1*^, Daniela Van Santen^2,9*^, Mirjam I Bakker ^2^, Judith Lammers^3^, Veronicah Mugisha^4^, Emmanuel Bagiruwigize^5^, Ludwig De Naeyer^1^, Anita Asiimwe^6^, Maarten F Schim van der Loeff^7,8,9^

**Affiliations:**

1. INTERACT, Kigali, Rwanda
2. KIT Biomedical Research, Royal Tropical Institute (KIT), Amsterdam, The Netherlands
3. Academic Medical Center (AMC), Amsterdam, the Netherlands
4. ICAP, Mailman School of Public Health, Columbia University, Kigali, Rwanda
5. Ruhengeri hospital, Ministry of Health, Kigali, Rwanda
6. University of Rwanda, Kigali, Rwanda
7. Amsterdam Institute of Global Health and Development (AIGHD), Academic Medical Center (AMC), Amsterdam, the Netherlands
8. Center for Infection and Immunity Amsterdam (CINIMA), AMC, Amsterdam, the Netherlands
9. Public Health Service of Amsterdam (GGD), Amsterdam, the Netherlands

* These authors contributed equally to this manuscript.

**This supplement contains four supplementary Tables (Tables S1 – S4) and two supplementary Figures (Fig S1 and Fig S2)**

| **Table S1. Characteristics of 65,716 clinic attendees of outpatient departments in eight health facilities by study phase and site, PITC study, Rwanda 2009-2010.** | | | | | | | | | | | | | | | | | | |
| --- | --- | --- | --- | --- | --- | --- | --- | --- | --- | --- | --- | --- | --- | --- | --- | --- | --- | --- |
|  | Rwaza | | Kinyinya | | Ruhengeri | | Muhoza | | Kibagabaga | | Kimironko | | Gasiza | | Kabuye | | Total | |
| **Total** | 5,924 | | 6,072 | | 9,088 | | 18,892 | | 5,596 | | 10,659 | | 5,470 | | 4,015 | | 65,716 | |
| **Phase 1** | No. | % | No. | % | No. | % | No. | % | No. | % | No. | % | No. | % | No. | % | No. | % |
| **Total** | 2,270 | 7.3% | 2,584 | 8.3% | 3,829 | 12.3% | 10,745 | 34.4% | 2,131 | 6.8% | 4,808 | 15.4% | 2,959 | 9.5% | 1,878 | 6.0% | 31,204 | 100.0% |
| **Sex** |  |  |  |  |  |  |  |  |  |  |  |  |  |  |  |  |  |  |
| Male | 895 | 39.5% | 1,127 | 43.7% | 1,572 | 47.3% | 3,188 | 29.7% | 830 | 39.0% | 1,895 | 39.4% | 891 | 30.2% | 781 | 41.7% | 11,179 | 36.5% |
| Female | 1,372 | 60.5% | 1,451 | 56.3% | 1,748 | 52.7% | 7,545 | 70.3% | 1,298 | 61.0% | 2,912 | 60.6% | 2,064 | 69.8% | 1,094 | 58.3% | 19,484 | 63.5% |
| Missing | 3 |  | 6 |  | 509 |  | 12 |  | 3 |  | 1 |  | 4 |  | 3 |  | 541 |  |
| **Median** **age (IQR)** | 32 | (23-50) | 29 | (23-40) | 34 | (25-48) | 27 | (20-40) | 27 | (22-37) | 26 | (22-32) | 30 | (23-45) | 29 | (23-40) | 28 | (22-41) |
| **Age group in years** | |  |  |  |  |  |  |  |  |  |  |  |  |  |  |  |  |  |
| 15 - 24 | 658 | 29.4% | 800 | 31.1% | 866 | 22.8% | 4,491 | 41.8% | 727 | 36.3% | 2,057 | 43.0% | 934 | 31.6% | 593 | 31.7% | 11,126 | 36.0% |
| 25 - 34 | 472 | 21.1% | 894 | 34.8% | 1,064 | 28.0% | 2,757 | 25.7% | 694 | 34.7% | 1,710 | 35.8% | 830 | 28.1% | 614 | 32.8% | 9,035 | 29.2% |
| 35 - 44 | 317 | 14.2% | 374 | 14.5% | 725 | 19.1% | 1,350 | 12.6% | 258 | 12.9% | 469 | 9.8% | 403 | 13.7% | 294 | 15.7% | 4,190 | 13.5% |
| ≥45 | 791 | 35.3% | 504 | 19.6% | 1,140 | 30.0% | 2,135 | 19.9% | 322 | 16.1% | 547 | 11.4% | 785 | 26.6% | 370 | 19.8% | 6,594 | 21.3% |
| Missing | 32 |  | 12 |  | 34 |  | 12 |  | 130 |  | 25 |  | 7 |  | 7 |  | 259 |  |
| **Phase 3** |  |  |  |  |  |  |  |  |  |  |  |  |  |  |  |  |  |  |
| **Total** | 3,654 | 10.6% | 3,488 | 10.1% | 5,259 | 16.9% | 8,147 | 23.6% | 3,465 | 10.0% | 5,851 | 17.0% | 2,511 | 7.3% | 2,137 | 6.2% | 34,512 | 100.0% |
| **Sex** |  |  |  |  |  |  |  |  |  |  |  |  |  |  |  |  |  |  |
| Male | 1,592 | 43.7% | 1,687 | 48.4% | 2,097 | 40.0% | 2,381 | 29.3% | 1,266 | 36.6% | 2,505 | 42.8% | 841 | 33.5% | 981 | 45.9% | 13,350 | 38.7% |
| Female | 2,052 | 56.3% | 1,800 | 51.6% | 3,152 | 60.0% | 5,748 | 70.7% | 2,191 | 63.4% | 3,341 | 57.2% | 1,670 | 66.5% | 1,155 | 54.1% | 21,109 | 61.3% |
| Missing | 10 |  | 1 |  | 10 |  | 18 |  | 8 |  | 5 |  | 0 |  | 1 |  | 53 |  |
| **Median age (IQR)** | 32 | (22-50) | 28 | (22-38) | 33 | (25-47) | 28 | (22-41) | 29 | (24-39) | 26 | (22-32) | 29 | (22-42) | 28 | (22-38) | 28 | (22-40) |
| **Age group in years** | |  |  |  |  |  |  |  |  |  |  |  |  |  |  |  |  |  |
| 15 - 24 | 1,279 | 35.2% | 1,282 | 36.8% | 1,106 | 22.9% | 2,943 | 36.2% | 963 | 28.0% | 2,471 | 42.5% | 833 | 33.2% | 740 | 34.6% | 11,617 | 34.2% |
| 25 - 34 | 659 | 18.1% | 1,088 | 31.2% | 1,510 | 31.3% | 2,342 | 28.8% | 1,307 | 38.0% | 2,190 | 37.7% | 755 | 30.1% | 694 | 32.5% | 10,545 | 31.0% |
| 35 - 44 | 493 | 13.6% | 507 | 14.6% | 870 | 18.0% | 1,166 | 14.3% | 525 | 15.3% | 594 | 10.2% | 349 | 13.9% | 328 | 15.4% | 4,832 | 14.2% |
| ≥45 | 1206 | 33.2% | 607 | 17.4% | 1,341 | 27.8% | 1,676 | 20.6% | 642 | 18.7% | 559 | 9.6% | 574 | 22.9% | 374 | 17.5% | 6,979 | 20.5% |
| Missing | 17 |  | 4 |  | 432 |  | 20 |  | 28 |  | 37 |  | 0 |  | 1 |  | 539 |  |

IQR interquartile range

**Table S2. Multivariable logistic regression to determine the association of study phase with HIV** testing rate **and HIV case finding in the outpatient department of 2 control health facilities in Rwanda, 2009-2010.**

|  | **HIV** testing rate |  |  |  |  | **HIV case finding** |  |  |  |
| --- | --- | --- | --- | --- | --- | --- | --- | --- | --- |
|  | **n/N (%)** |  | **aOR** | **(95% CI)** | **P** | **n/N (%)** | **aOR** | **(95% CI)** | **P** |
| **Study phase** |  |  |  |  | <0.001 |  |  |  | 0.128 |
| Phase 1 | 242/4,837 (5.0%) |  | 1 |  |  | 9/4,837 (0.19%) | 1 |  |  |
| Phase 3 | 627/4,648 (13.5%) |  | 2.76 | (2.34-3.24) |  | 17/4,648 (0.37%) | 1.85 | (0.83-4.12) |  |
| **Study site** |  |  |  |  | <0.001 |  |  |  | 0.011 |
| Gasiza | 63/5,470 (1.2%) |  | 1 |  |  | 8/5,470 (0.15%) | 1 |  |  |
| Kabuye | 806/4,015 (20.1%) |  | 20.44 | (15.71-26.60) |  | 18/4,015 (0.45%) | 2.86 | (1.27-6.41) |  |
| **Sex** |  |  |  |  | 0.062 |  |  |  | 0.825 |
| Females | 463/5,983 (7.7%) |  | 1 |  |  | 15/5,983 (0.25%) | 1 |  |  |
| Males | 405/3,494 (11.6%) |  | 1.16 | (0.99-1.34) |  | 11/3,494 (0.31%) | 1.09 | (0.51-2.34) |  |
| **Age group (years)** |  |  |  |  | 0.358 |  |  |  | 0.008 |
| 15 - 24 | 304/3,100 (9.8%) |  | 1 |  |  | 3/3,100 (0.10%) | 1 |  |  |
| 25 - 34 | 286/2,893 (9.9%) |  | 0.99 | (0.82-1.18) |  | 8/2,893 (0.28%) | 2.83 | (0.74-10.85) |  |
| 35 - 44 | 136/1,374 (9.9%) |  | 0.99 | (0.78-1.24) |  | 10/1,374 (0.73%) | 7.51 | (2.05-27.60) |  |
| ≥45 | 143/2,103 (6.8%) |  | 0.83 | (0.66-1.03) |  | 5/2,103 (0.24%) | 2.77 | (0.65-11.88) |  |

Abbreviations: aOR: adjusted Odds ratio; CI: confidence interval.

Information on age was missing from 15 attendees and information on sex from 8 attendees.

Adjusted odds ratios are adjusted for all other variables.

**Table S3. Characteristics of 77,389 clinic attendees of outpatient and voluntary counseling and testing departments in eight health facilities by study phase and site, PITC study, Rwanda 2009-2010.**

|  | **Rwaza** | | **Kinyinya** | | **Ruhengeri** | | **Muhoza** | | **Kibagabaga** | | **Kimironko** | | **Gasiza (c)** | | **Kabuye (c)** | | **Total** | |  |
| --- | --- | --- | --- | --- | --- | --- | --- | --- | --- | --- | --- | --- | --- | --- | --- | --- | --- | --- | --- |
| **Total** | 6,850 | | 6,950 | | 9,088 | | 23,833 | | 6,004 | | 11,431 | | 6,756 | | 6,477 | | 77,389 | |  |
| **Phase 1** | No. | % | No. | % | No. | % | No. | % | No. | % | No. | % | No. | % | No. | % | No. | % | |
| **Total** | 2,863 | 7.6% | 3,072 | 8.1% | 3,829 | 10.1% | 13,614 | 36.1% | 2,381 | 6.3% | 5,155 | 13.7% | 3,602 | 9.5% | 3,231 | 8.6% | 37,747 | 100.0% | |
| **Sex** |  |  |  |  |  |  |  |  |  |  |  |  |  |  |  |  |  |  | |
| Male | 1172 | 41.0% | 1,439 | 46.9% | 1,572 | 47.3% | 4,137 | 30.4% | 931 | 39.2% | 2,063 | 40.0% | 1170 | 32.5% | 1432 | 44.4% | 13,916 | 37.4% | |
| Female | 1,688 | 59.0% | 1,627 | 53.1% | 1,748 | 52.7% | 9,465 | 69.6% | 1,446 | 60.8% | 3,090 | 60.0% | 2,428 | 67.5% | 1,795 | 55.6% | 23,287 | 62.6% | |
| Missing | 3 |  | 6 |  | 509 |  | 12 |  | 4 |  | 2 |  | 4 |  | 4 |  | 544 |  | |
| **Median age (IQR)** | 32 | (22-48) | 30 | (23-41) | 34 | (25-48) | 27 | (21-38) | 27 | (22-36) | 26 | (22-32) | 29 | (22-43) | 28 | (23-38) | 28 (22-40) |  | |
| **Age group in years** | |  |  |  |  |  |  |  |  |  |  |  |  |  |  |  |  |  | |
| 15 - 24 | 943 | 33.4% | 905 | 29.6% | 866 | 22.8% | 5,607 | 41.2% | 841 | 37.4% | 2,187 | 42.6% | 1,251 | 34.8% | 1,049 | 32.5% | 13,649 | 36.4% | |
| 25 - 34 | 596 | 21.1% | 1060 | 34.6% | 1,064 | 28.0% | 3,890 | 28.6% | 798 | 35.5% | 1,857 | 36.2% | 996 | 27.7% | 1,162 | 36.1% | 11,423 | 30.5% | |
| 35 - 44 | 393 | 13.9% | 467 | 15.3% | 725 | 19.1% | 1,785 | 13.1% | 275 | 12.2% | 511 | 10.0% | 502 | 14.0% | 489 | 15.2% | 5,147 | 13.7% | |
| ≥45 | 895 | 31.7% | 628 | 20.5% | 1,140 | 30.0% | 2,318 | 17.0% | 334 | 14.9% | 574 | 11.2% | 846 | 23.5% | 523 | 16.2% | 7,258 | 19.4% | |
| Missing | 36 |  | 12 |  | 34 |  | 14 |  | 133 |  | 26 |  | 7 |  | 8 |  | 270 |  | |
| **Phase 3** |  |  |  |  |  |  |  |  |  |  |  |  |  |  |  |  |  |  | |
| **Total** | 3,987 | 10.1% | 3,878 | 9.8% | 5,259 | 13.9% | 10,219 | 25.8% | 3,623 | 9.1% | 6,276 | 15.8% | 3,154 | 8.0% | 3,246 | 8.2% | 39,642 | 100.0% | |
| **Sex** |  |  |  |  |  |  |  |  |  |  |  |  |  |  |  |  |  |  | |
| Male | 1,729 | 43.5% | 1,893 | 48.8% | 2,097 | 40.0% | 3,297 | 32.3% | 1,338 | 37.0% | 2,729 | 43.5% | 1,099 | 34.8% | 1,544 | 47.6% | 15,726 | 39.7% | |
| Female | 2248 | 56.5% | 1984 | 51.2% | 3152 | 60.0% | 6,903 | 67.7% | 2,277 | 63.0% | 3,542 | 56.5% | 2,055 | 65.2% | 1,701 | 52.4% | 23,862 | 60.3% | |
| Missing | 10 |  | 1 |  | 10 |  | 19 |  | 8 |  | 5 |  | 0 |  | 1 |  | 54 |  | |
| **Median age (IQR)** | 30 | (22-49) | 28 | (22-37) | 33 | (25-47) | 27 | (22-39) | 29 | (24-38) | 26 | (22-32) | 28 | (22-40) | 28 | (23-36) | 28 (22-39) |  | |
| **Age group in years** | |  |  |  |  |  |  |  |  |  |  |  |  |  |  |  |  |  | |
| 15 - 24 | 1,444 | 36.4% | 1,434 | 37.0% | 1,106 | 22.9% | 3,861 | 37.9% | 1022 | 28.4% | 2,651 | 42.5% | 1,113 | 35.3% | 1,107 | 34.1% | 13,738 | 35.1% | |
| 25 - 34 | 755 | 19.0% | 1,255 | 32.4% | 1,510 | 31.3% | 3,128 | 30.7% | 1,388 | 38.6% | 2,384 | 38.2% | 966 | 30.6% | 1,190 | 36.7% | 12,576 | 32.2% | |
| 35 - 44 | 522 | 13.2% | 558 | 14.4% | 870 | 18.0% | 1,400 | 13.7% | 536 | 14.9% | 630 | 10.1% | 442 | 14.0% | 500 | 15.4% | 5,458 | 14.0% | |
| ≥45 | 1248 | 31.4% | 627 | 16.2% | 1,341 | 27.8% | 1,807 | 17.7% | 647 | 18.0% | 574 | 9.2% | 633 | 20.1% | 447 | 13.8% | 7,324 | 18.7% | |
| Missing | 18 |  | 4 |  | 432 |  | 23 |  | 30 |  | 37 |  | 0 |  | 2 |  | 546 |  | |

IQR=Interquartile range. (c)= control site.

**Table S4. Multivariable logistic regression to determine the association of study phase with HIV** testing rate **and HIV case finding in the outpatient and voluntary counseling and testing departments of 2 control health facilities in Rwanda, 2009-2010.**

|  | **N** | **% HIV** testing rate | **aOR** | **95% CI** | **P** | **% HIV**  **positive** | **aOR** | **95% CI** | **P** |
| --- | --- | --- | --- | --- | --- | --- | --- | --- | --- |
| **Study phase** |  |  |  |  |  |  |  |  |  |
| Phase 1 | 6,833 | 32.8% | 1 |  | <0.001 | 0.75% | 1 |  | 0.175 |
| Phase 3 | 6,400 | 37.2% | 1.14 | 1.06-1.24 |  | 1.02% | 1.29 | 0.89-1.86 |  |
| **Study site** |  |  |  |  |  |  |  |  |  |
| Gasiza | 6,756 | 20.0% | 1 |  | <0.001 | 0.31% | 1 |  | <0.001 |
| Kabuye | 6,477 | 50.5% | 3.85 | 3.56-4.16 |  | 1.47% | 4.35 | 2.73-6.92 |  |
| **Sex** |  |  |  |  |  |  |  |  |  |
| Females | 7,979 | 30.8% | 1 |  | <0.001 | 0.73% | 1 |  | 0.213 |
| Males | 5245 | 41.1% | 1.37 | 1.27-1.48 |  | 1.11% | 1.26 | 0.88-1.81 |  |
| **Age group (years)** |  |  |  |  |  |  |  |  |  |
| 15 - 24 | 4,520 | 38.1% | 1 |  | <0.001 | 0.42% | 1 |  | <0.001 |
| 25 - 34 | 4,314 | 39.6% | 0.97 | 0.88-1.06 |  | 1.07% | 2.34 | 1.37-4.01 |  |
| 35 - 44 | 1,933 | 36.0% | 0.85 | 0.76-0.96 |  | 1.97% | 4.56 | 2.62-7.93 |  |
| ≥45 | 2,449 | 20.0% | 0.42 | 0.37-0.47 |  | 0.53% | 1.43 | 0.71-2.90 |  |
| Abbreviations: aOR: adjusted Odds ratio; CI: confidence interval. Information on age was missing from 17 attendees and information on sex from 9 attendees. | | | | | | | | | |

**Figure S1. HIV** testing rate **and testing cascade in OPD in phase 3 at intervention sites, Rwanda, 2009-10.**

**
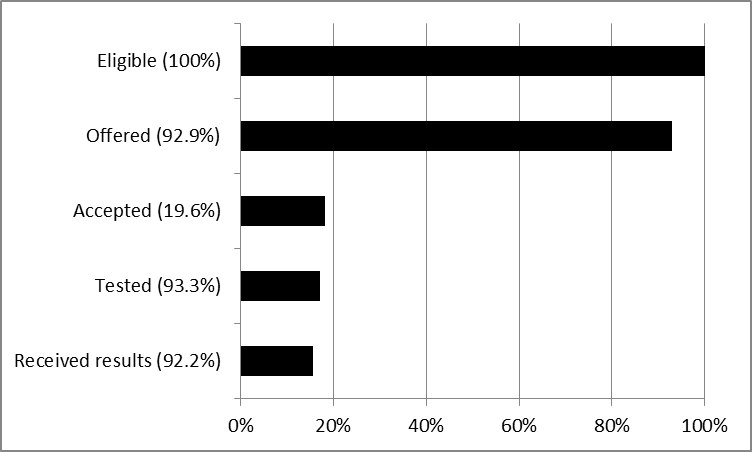
**

**Figure S2: Reasons for refusing an HIV test in phase 3 at intervention sites, Rwanda, 2009-10.**

**
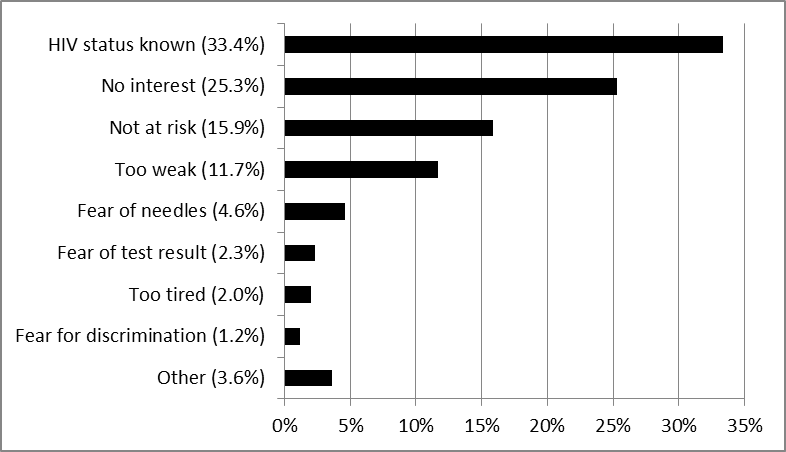
**
